# The Early Diagnosis of Uterine Cancer

**Published:** 1909-05-15

**Authors:** 


### The Hospital

A Journal of

The Medical Sciences and Hospital Administration.

New Series. No. 115, Vol. V. [No. 1187, Vol. XLVI.] SATURDAY, MAY 15, 1909.

### The Early Diagnosis of Uterine Cancer.

We owe it to the courtesy of the Editor of the *British Medical Journal*, who has forwarded advance proofs, that we are enabled this week to print in outline the report of the Committee appointed by the British Medical Association to consider how best to secure the earlier diagnosis and treatment of uterine cancer. The report contains two appeals which it is desired to circulate as widely as possible—one to members of the medical profession, the other to midwives and nurses. The first of these two is very much the longer, and is, in fact, a *résumé* of the pathology, symptoms, and signs of malignant disease of the uterus. It is drawn up with great care, and it is very well worthy of the closest study by everyone whose advice is ever asked by women about their health, whether he be a general practitioner, a gynæcologist, or a specialist in any other branch of medicine or surgery.

On reading through this document, among the first impressions gained is one almost of humiliation that it should be necessary in the twentieth century to point out to qualified men their bounden duty to make a physical examination of those of their patients whose symptoms are suggestive of malignant disease. It is not to be doubted that the members of the committee felt strongly the importance of overcoming the laxity which it is to be admitted does exist in the profession with regard to this matter. In point of fact, the whole attitude of the surgeon towards malignant disease of all kinds, not of the uterus alone, has been changing rapidly of late years. The routine use of the microscope in diagnosis, the steady progress in surgical technique, and the resulting greatly improved ultimate results of operation are practically the product of the last decade or so; and the very remarkable success which now attends operation on early cases serves by contrast to illuminate the still dismal record of those in which the disease has gone too long unchecked. The cry of the surgeon is for early cases, and for the education of the practitioner in detecting them and advising operation. For all that it is still true that even now many of our standard text-books contain descriptions of malignant disease in various situations which are essentially pictures of advanced cases. Cachexia, palpably enlarged glands, fixity of the growth, dimpling of the skin, and many other signs are still given in surgical works as points to be observed in the differential diagnosis of cancerous tumours. It is on the earliest, not the latest, signs that stress should be laid; the object aimed at should be the diagnosis, not of a tumour which is indisputably malignant, but of one to be reasonably regarded with suspicion sufficient to justify its exploration or its submission to the miscroscope of the pathologist.

Granted, then, that just at present the diagnosis of malignant disease is, to a certain extent, a reproach to the profession, though beyond question less so every year. The situation must clearly be amended. The expert in fevers who sees a faucial inflammation of suspicious aspect does not wait until he has cultivated the bacillus diphtheriæ before injecting antitoxin; why should the medical man confronted by a symptom possibly due to a much more terrible disease—carcinoma of the uterus—refrain from diagnosis and treatment until the first is obvious and the second impossible? The answer, of course, is that his patients are not so enthusiastic about a pelvic examination as of an inspection of their fauces; at least, no other of any weight suggests itself. Now this objection, due to quite natural feminine reluctance, is at best an excuse, not a valid reason; and the doctor who knows that he ought to make a vaginal examination but refrains from doing so on this ground is one whose failure of an obvious duty might well be described in very harsh terms. Moreover, as the memorandum of the British Medical Association's Committee points out, a frank explanation of the conscientious necessity which compels the step in question will almost always suffice to remove the objection felt or expressed.

So much, then, for the improved education of the medical profession, which the publication of the report will, we feel sure, do much to promote. There remain two other important factors—one the education of the public itself, the other that of midwives and nurses, who are so often taken into confidence long before any appeal is made to a medical man or woman. With the education of the public the present report does not deal, but it may be remarked that it is of importance subsidiary to that of midwives and doctors. Already it is very noticeable how much less the fear of "the knife" permeates the lay population than it formerly did—a natural consequence of the advances made in surgery during the last generation. The appeal issued to nurses and midwives is most commendably brief and pointed, as it must be if it is to impress itself firmly on the minds of those to whom it is addressed. If there is one point connected with these excellent memoranda which is in the least degree capable of improvement, it is the arrangement which places first among the early signs of cancer of the womb "*Bleeding*, which occurs after the change of life." It is true that No. 3 explains that intermenstrual bleeding in younger women is also of importance; but experience proves that to start off with the statement we have quoted may by many be too hastily interpreted into a significance quite erroneous, to the effect that hæmorrhage before the menopause is of no consequence. The emphasis laid on the paragraph which impresses on those to whom it is addressed that pain, wasting, foul discharge, and profuse bleeding are often all four absent in cases of cancer still in the curable stage, is not a whit too great. The dissemination of this appeal throughout the medical and nursing Press is a project which must have the best wishes of everyone who is interested in the crusade against cancer.

